# Mitochondrial genomes of the South American electric knifefishes (Order Gymnotiformes)

**DOI:** 10.1080/23802359.2016.1174090

**Published:** 2016-06-20

**Authors:** Ahmed A. Elbassiouny, Ryan K. Schott, Joseph C. Waddell, Matthew A. Kolmann, Emma S. Lehmberg, Alexander Van Nynatten, William G. R. Crampton, Belinda S. W. Chang, Nathan R. Lovejoy

**Affiliations:** a Department of Biological Sciences, University of Toronto Scarborough, Toronto, ON, Canada;; b Department of Ecology and Evolutionary Biology, University of Toronto, Toronto, ON, Canada;; c Department of Biology, University of Central Florida, Orlando, FL, USA;; d Department of Cell and Systems Biology, University of Toronto, Toronto, ON, Canada

**Keywords:** Gymnotiformes, knifefish, phylogeny

## Abstract

Three complete mitochondrial genomes of South American electric fishes (Gymnotiformes), derived from high-throughput RNA sequencing (RNA-Seq), are reported herein. We report the complete mitochondrial genome of the bluntnose knifefish *Brachyhypopomus* n.sp. VERD, determined from newly sequenced data. We also provide the complete mitochondrial genomes for *Sternopygus arenatus* and the electric eel *Electrophorus electricus*, assembled from previously published transcriptome data. The mitochondrial genomes of *Brachyhypopomus* n.sp. VERD*, Sternopygus arenatus* and *Electrophorus electricus* have 13 protein-coding genes, 1 D-loop, 2 ribosomal RNAs and 22 transfer RNAs, and are 16,547, 16,667 and 16,906 bp in length, respectively. Phylogenetic analysis of the eight available mitochondrial genomes of gymnotiform fishes shows *Apteronotus* to be the sister lineage of other gymnotiformes, contradicting the “Sinusoidea” hypothesis that Apteronotidae and Sternopygidae are sister taxa.

The Gymnotiformes are a diverse group of more than 200 species of neotropical ostariophysan fishes that produce electric signals for both navigation and communication. The species of this order have been used as models in neuroscience, including studies of electrosensory modulation (Márquez et al. 2013) and central nervous system regeneration (Zupanc & Sîrbulescu 2013). To date, complete mitochondrial genomes have been reported for the gymnotiforms *Apteronotus albifrons*, *Brachyhypopomus occidentalis*, *Gymnorhamphichthys* sp. and *Eigenmannia* sp., and near-complete mitochondrial genomes have been reported for *Gymnotus carapo* and *Electrophorus electricus* (Nakatani et al. 2011). Here, we report the complete mitochondrial genome for the undescribed species *Brachyhypopomus* n.sp. VERD (Crampton et al. unpublished) which we assembled from newly generated RNA-seq data. We also report the complete mitochondrial genomes for *Sternopygus arenatus* and *Electrophorus electricus*, based on assemblies of RNA-seq data produced by Gallant et al. (2014).

Electric organ was collected from a fresh specimen of *Brachyhypopomus* n.sp. VERD in the field (voucher specimen MUSM 54490, 04°53.986′ S, 073°38.849′ W) and preserved in RNAlater (Qiagen, Hilden, Germany). The electric organ was homogenized in Trizol (Invitrogen, Carlsbad, CA) using a BeadBug (Benchmark Scientific, Edison, NJ). Total RNA was extracted following a combined Trizol/RNeasy (Qiagen) protocol according to the manufacturer’s instructions. Library construction and sequencing on the Illumina HiSeq pipeline were performed at The Centre for Applied Genomics, the Hospital for Sick Children (Toronto). *Sternopygus* and *Electrophorus* raw reads were obtained from the NCBI SRA repository (SRR1299088, SRR1299078 and SRR1299082). For all datasets, sequencing reads were trimmed with Trimmomatic v0.33 (Bolger et al. 2014) using default settings. Trimmed reads were mapped to the phylogenetically closest mitochondrial genome currently available on Genbank using the map-to-reference feature of Geneious v6 (Biomatters, Aukland, New Zealand; Kearse et al. 2012): *Brachyhypopomus* n.sp. VERD reads were mapped to *B. occidentalis* (Genbank AP011570), *Sternopygus arenatus* to *Eigenmannia* sp. (Genbank AB054131) and *Electrophorus electricus* to the near-complete mitochondrial genome of *E. electricus* (Genbank AP011978.1). Consensus sequences of aligned reads were generated using the highest quality base calls. The assembled mitochondrial genomes were annotated using MitoAnnotator (Iwasaki et al. 2013).

To verify the species identities for both published and newly produced gymnotiform mitochondrial genomes, we compared the cytochrome b (*cytb*) and cytochrome oxidase I (*coI*) genes from the mitochondrial genomes to available sequences in Genbank, as well as a large database of *cytb* and *coI* sequences produced in the course of an ongoing multi-gene phylogenetic analysis of gymnotiform fishes (Janzen et al. unpublished). We confirmed the species identity of the mitochondrial genomes from *Apteronotus albifrons*, *Brachyhypopomus occidentalis*, *Gymnotus carapo* and *Electrophorus electricus*. We were unable to determine the species identity of *Gymnorhamphichthys* sp. or *Eigenmannia* sp. due to an absence of high sequence similarity matches with Genbank sequences or sequences in our database, but we did confirm the generic identity of these samples. Interestingly, our analysis suggests that the transcriptome reported from *Sternopygus macrurus* (SRR1299088) is actually from *Sternopygus arenatus*. The *cytb* and *coI* sequences from the mitochondrial genome assembled from this transcriptome data match *S. arenatus* with 99.8% sequence identity, but are only 90.6% identical to *S. macurus*. Phylogenetic analysis (not shown) using a dataset of Sternopygidae species (Maldonado-Ocampo 2011) indicate that the transcriptome data clusters with known *S. arenatus* sequences. Finally, we determined the nuclear *zic1* sequence from the transcriptome and compared it to *zic1* species from multiple sternopygid species, which confirmed the identity of this sample as *S. arenatus*.

The mitochondrial genomes of *Brachyhypopomus* n.sp. VERD (Genbank KX058570), *Sternopygus arenatus* (Genbank KX058571) and *Electrophorus electricus* (Genbank KX058572) are complete, with 13 protein-coding genes, 1 D-loop, 2 ribosomal RNAs and 22 transfer RNAs for a total length of 16,547, 16,667 and 16,906 bp, respectively. Base compositions are typical for vertebrate mitochondrial genomes with 28.7% A, 29.9% C, 15.7% G, 25.7% T for *Brachyhypopomus* n.sp. VERD, 31.3% A, 28.5% C, 14.6% G, 25.5% T for *Sternopygus arenatus*, and 31.3% A, 26.0% C, 13.6% G, 29.1% T, 39.6% for *Electrophorus electricus*.

To produce a phylogenetic tree using gymnotiform mitogenomes, we aligned the eight gymnotiform mitochondrial genomes with available Characiformes and Siluriformes and a cypriniform outgroup (*Zacco platypus*) using MAFFT (Katoh & Standley 2013). We then generated a maximum likelihood phylogeny using IQ-Tree (Nguyen et al. 2015) with 1000 bootstrap replicates (Figure 1). We tested for the best-fit model based on Akaike Information Criterion (AIC), corrected Akaike Information Criterion (AICc) and Bayesian Information Criterion (BIC), which converged on the GTR + I + G4 model.

**Figure 1.**
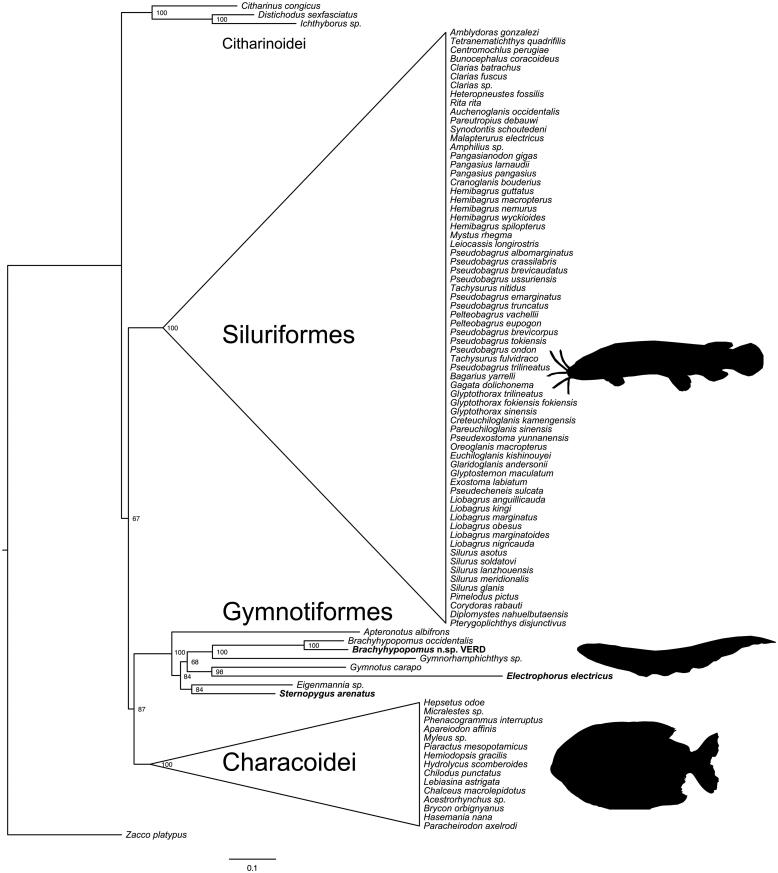
Phylogenetic relationships of gymnotiform electric knifefishes and allies based on maximum likelihood analysis of mitochondrial genomes. Numbers at nodes show bootstrap proportions. Mitochondrial genomes assembled for this study are in bold.

Our phylogenetic reconstruction confirms the monophyly of Siluriformes and Gymnotiformes, but indicates that Characiformes are non-monophyletic and are divided into Characoidei and Citharinoidei, as seen in other molecular studies (Nakatani et al. 2011; Chen et al. 2013), and in contrast to morphological evidence (see summary in Chen et al. 2013). Within Gymnotiformes, patterns of relationships largely match expectations based on taxonomy and prior phylogenetic studies. The two *Brachyhypopomus* species are sister taxa, and are most closely related to *Gymnorhamphichthys*, supporting a close relationship between the families Hypopomidae and Rhamphichthyidae (Maldonado-Ocampo et al. 2014). *Gymnotus* and *Electrophorus* are well-supported as sister taxa, corroborating the long-standing hypothesis that groups these genera in the family Gymnotidae (Albert & Crampton 2005; Lovejoy et al. 2010). *Sternopygus* and *Eigenmannia*, both members of the family Sternopygidae, group together as expected. The earliest diverging gymnotiform lineage has not been decisively determined. In this study, *Apteronotus* is sister to the other gymnotiform lineages, matching the pattern based on sodium channel sequences (Arnegard et al. 2010), but in contrast to other molecular and morphological studies that place Gymnotidae at the base of the gymnotiform tree (Albert & Crampton 2005; Tagliacollo et al. 2016). This topology contradicts a hypothesis that groups as sister taxa Sternopygidae and Apteronotidae, the families with wave-type electric organ discharges, in a “Sinusoidea” clade (Albert 2001).

The results reported here contribute to ongoing efforts to reconstruct teleost phylogenies based on mitochondrial genomes, and also highlight the value of mitochondrial genes as markers for confirming species identifications for genomic resources.
